# Extract from a Report of Diseases Prevalent in the Garrison of St. Vincent, in 1824, Containing an Account of an Abdominal Abscess, in Which Pus Was Voided through the Urethra

**Published:** 1824-10

**Authors:** J. Ellick

**Affiliations:** Surgeon to the Forces.


ART. II.—*Extract from a Report of Diseases prevalent in the Garrison of St. Vincent, in* 1824, *containing an Account of an Abdominal Abscess, in which Pus was voided through the Urethra.* By J. ELLICK, Esq. Surgeon to the Forces.

I CANNOT, in justice to the science of surgery, conclude this Report, without mentioning a singular case of abdominal abscess, which lately occurred in one of my own children, a boy between seven and eight years of age. About the middle of November last, he was observed to be a little lame of his right leg, but he did not complain of any pain or uneasiness: in the course of some days it increased, and, on examination, I found some hardness and tenderness on making pressure just above Poupart's ligament, on the outer side of the great vessels going to the thigh. As I found he had been of late running about in the wet, and sitting occasionally on the damp grass before the house, I became fearful there was some incipient affection of the hip-joint; and therefore enjoined rest, and applied leeches to the groin. He gradually got worse; lost his appetite, became drowsy, feverish, and emaciated, with profuse sweating at night. By the middle of December, he could not lift his foot from the ground without supporting the ham with his hand, and was obliged, when at rest, to bend his thigh forward at a right angle with the body; the tenderness in the groin was increased on pressure, but no appearance of a collection of matter could be ascertained. The thigh could be rotated with ease, and could bear pressure upwards; which was in favour of the hip-joint not being affected. He still got worse daily; became almost reduced to a skeleton; and I was much alarmed for his safety. He continued drowsy, and slept much, occasionally moaning and starting in his sleep at night, but did not complain of pain, unless the limb was moved or the groin pressed upon. I never observed that he had any rigors.

On the 28th of December, while resting on the sofa in an adjoining room, he suddenly called to me, and said he had a strong inclination to make water. I held the utensil, and, to my surprise, he voided between two and three ounces of pure pus from the urethra, which was followed by the urine: he expressed that he voided it with ease, and, in his own words, "comfortably;" saying that, for some time previous, he had always felt pain in making water. He had a good night; felt better in every way.

By the following morning, he passed about the same quantity of pus with his urine, which I measured after its deposit. He ate a hearty breakfast, and continued in better spirits through the day. He seemed to improve, though but slowly, for some days; and the pus in the urine gradually decreased in quantity.

On the 5th, 6th, and 7th of January, the urine was clear, and did not contain any purulent matter. On the 8th, there was some tumefaction and hardness of the abdomen, felt along the direction of an horizontal line, drawn from the ilium to midway between the umbilicus and pubis, and from thence a little downwards towards the pubis; and which gave reason to suppose (particularly as, near the extremity of the line next to the ilium, the part was somewhat softer to the feel,) that an abscess was pointing in that direction. He then had little or no fever, but still was much emaciated. In the course of another week, the tumor was completely developed, and, from the elasticity of its feel, evidently contained pus, and showed a disposition to open at a short distance from the extremity of the line before mentioned, near to the ilium, above Poupart's ligament. Of late, his health, spirits, and appetite, had evidently improved; but the emaciation of body continued.

On the nights of the 17th and 18th, he became very restless, sweated much, seemed greatly oppressed, and complained of much pain in the ham and calf of the leg of the affected side; and consequently, being myself apprehensive that there might not be sufficient energy in his constitution, which was apparently sinking, to cause a natural evacuation of the contents of the abscess, and that it was about to take some other internal route, I was determined, early in the morning of the 19th, to make an external opening, and evacuate the matter; which I accordingly did, with a common lancet, making an opening about the size of that generally done in bleeding from a large orifice, with the view of allowing the matter to discharge itself gradually, and not expose the cavity. About four ounces of pure healthy pus was immediately evacuated, and a good deal more oozed from the opening afterwards. A small poultice was applied over the part only where the abscess had pointed.

The abscess continued to discharge freely; and although not much amendment in his general health was observed during the first week after it was opened, yet subsequently he recovered rapidly, and almost unexpectedly. In the course of fifteen days, the external wound healed, leaving some hardness along the line from the ilium to midway between the umbilicus and pubis.

The discharge during the second week became serous, and the urine was a little turbid,—more, apparently, from albuminous than purulent matter. The thigh gradually returned to its natural straight position, and its movements became performed with freedom. His health, appetite, and spirits, rapidly improved; he gained flesh; and at this period (March 20th) is perfectly recovered, and looks better than he has ever done since his arrival in the West Indies, more than three years ago.

In regard to the cause of the formation of this abscess, which perhaps by some would have been denominated *psoas*, I have some reason to think it was owing to accident. He acknowledged that, just before he first had any degree of lameness, he received a kick from another boy on the lower part of the abdomen, and also that he had been suddenly tripped up by another. His constitution is naturally a good one, though somewhat impaired by a West-India climate; he has never had any symptom of a scrofulous nature, nor has any thing of the kind ever been known to exist in any of the family. For some time before his illness, he was somewhat out of health: he looked thin, and his belly was a little tumid; which was attributed to his running about in the wet, and occasionally exposing himself to the heat of the sun. From whatever cause it originated, whether accidental or constitutional, I consider the case, with its fortunate termination, to be one of rare occurrence; and therefore think it worthy of occupying a place in the Report.

He was seen occasionally, during the progress of the disease, by Mr. Blake, surgeon of the 5th regiment, and Mr. Mac Gibbon, hospital-assistant. The former gentleman, who stands high in professional acquirements, was persuaded in his own mind that it was a regular psoas abscess; and evinced apprehension of a fatal result speedily following the opening of the abscess, from the extreme degree of emaciation to which my little patient was reduced.

*St. Vincent; March 20th*, 1824.

